# Supplementary material for: Patterns of Intron Gain and Loss in Fungi
Source: PLoS Biol. 2004 Nov 30;2(12):e422. doi: 10.1371/journal.pbio.0020422 (PMC532390; doi:10.1371/journal.pbio.0020422)
Supplement: Table S1 — Also available at http://genes.mit.edu/NielsenEtAl/. (4.3 MB ZIP). [file pbio.0020422.st001.zip › NielsenEtAl/html/383.html]

AN8225.1.NCU08893.1.MG05957.1.FG05932.1


```
 CLUSTAL W (1.82) Multiple Sequence Alignments - Introns Inserted


Sequence 1: NCU08893.1	303 aa
Sequence 2: MG05957.1	298 aa
Sequence 3: FG05932.1	301 aa
Sequence 4: AN8225.1	310 aa
Alignment Length: 312 aa
Number Identitical Residues: 81 aa
Alignment Score (without introns) 5163


MG05957.1 	-------MASTQQCLASLARLSIRP--ATRPLATSVPRFLVPSVA--QPRFASVNKD---
NCU08893.1	-------MASTQQCLASLARLSLST--PTRAALPTIPKFLVPSVAASQVRYATNNPNKGG
FG05932.1 	-------MAPIDRCLASMARLSLLQ--TSRPAIPSIPKFLAPAAAQ-QVRQASVVRIRKT
AN8225.1  	MSSFSALMPTGARALLSICRPSIRQGSMAAPLFFGYQQAQARGAATARNKQAAERRKRDK
          	 :: :: *..  :.* *:.* *:  .: : .      :  . ..* :: : *:       

MG05957.1 	PKKKEKKKKELKQYRVHRKDTLLTFSLCDAIR2YIRAFEVGKPPGSVKYELAVKLTTNKR
NCU08893.1	AKKAPKKKKQYKFFKSWDLTGQQQFSLCDAMR2YLRAVEVGQPPLSVKYEVHVKLRTKKN
FG05932.1 	AKKKKPLPKDFKRHNLNKRE-FPQYSLCEAMR2ILRAVEVGEPPASIKYELHINLKTARN
AN8225.1  	AAKSAKSKKGPREFKQKDLKDVEQFALCDAMR2YLRAFEVGREPAVSKYEVHIRLKTRRD
          	. *     *  : ..         ::**:*:*  :**.***. *   ***: :.* * : 

MG05957.1 	SAIVRSRVRLPHSVRSDIRVAVICREGSLVATEARMAGAVATGEETLFQQIKDGNIDFNR
NCU08893.1	GPVVRDRVRLPTPVKTDTRIAVICPEGSALQEEAKNLGAVMAGEETLFEAIRSGNFPFNK
FG05932.1 	GPVIKNSVRLPHPVQSDWQIAVVCPEGSEIARDATAAGAVAVGQETLFEAIRQEKINFDR
AN8225.1  	GPVIRNMIRFPHSVQTESRICVICPPGSKHAKDALAAGAVLVGEDEVFEVVKSGRIEFDR
          	..:::. :*:* .*::: ::.*:*  **    :*   *** .*:: :*: ::. .: *::

MG05957.1 	LICHSDSLGALNKANLGRILGPKGLMPSDKTKTVTKDVKGLLRDLSGAEDYRERDGVVRM
NCU08893.1	LLCHTESEGALRKANVGKLLGPKGLMPSGKTKTITNNLEATFRDMIGMDEYRERNGVVRM
FG05932.1 	LICHEASEAALNKAGLGKILGPKGLMPSKRMRTIVPDVVKSMRDSAGAADYRERQGVIRL
AN8225.1  	CLAHPDSMPALAKAGLGRILGPRGLMPSAKTGTVVEDVAARVEMLRGGTVYRERDAVVRL
          	 :.*  *  ** **.:*::***:***** :  *:. ::   ..   *   ****:.*:*:

MG05957.1 	PIGQLTFTPEQLSDNIKAFVDALKKDIAKLESQVVKTIDEI~VLSTTHAPGFSLNGGFRS
NCU08893.1	AVGQLGFTPKQLAENIRVFMAKIKSDIGKLDDTTPKMVEEV~VLSTTHGPGMSLNAEFAP
FG05932.1 	AIGQLGYSPDQLKNNIKVLLAKIKAECTEISEEVHKEVHEV~ILSTTHGPGISLNGNLKD
AN8225.1  	PIGQLGFSPEQLRDNLRVTIDQVKKDAASLNDRITKEIYEV0VLSSTHGPGFSLNGEFK-
          	.:*** ::*.** :*::. :  :* :  .:..   * : *: :**:**.**:***. :  

MG05957.1 	TDENLTTAQLTGPM
NCU08893.1	TDDKIKPEDLESVM
FG05932.1 	AEEKITPEALSSIM
AN8225.1  	TENSVDPALLTGP-
          	:::.: .  * .
```
